# Antifungal Effect of the Proteolytic Fraction P1G10 Stabilized by Alginate–Chitosan Polyelectrolyte Complexation Against *Botrytis cinerea*

**DOI:** 10.3390/foods15101723

**Published:** 2026-05-14

**Authors:** Jonathan Cisternas-Jamet, Verónica Plaza, María José Torres-Ossandón, Carlos Salas, Claudia Bernal, Luis Castillo

**Affiliations:** 1 Departamento de Ciencias Básicas, Facultad de Ciencias, Universidad Santo Tomás, Panamericana Norte 1068, La Serena 1700000, Chile; jonathancisternasja@santotomas.cl; 2 Centro de Investigación y Modelación de Negocios CIMON, Universidad Santo Tomás, Panamericana Norte 1068, La Serena 1700000, Chile; 3 Laboratorio de Bioquímica y Biología Molecular, Departamento de Biología, Universidad de La Serena, Av. Raúl Bitrán 1305, La Serena 1700000, Chile; vplaza@userena.cl; 4 Departamento de Ingeniería en Alimentos, Universidad de La Serena, Av. Raúl Bitrán 1305, La Serena 1700000, Chile; 5 Departamento en Ciencia y Tecnología de los Alimentos, Facultad Tecnológica, Universidad de Santiago de Chile, Santiago 9170124, Chile; mtorresossandon@gmail.com; 6 Instituto de Ciencias Biológicas, Universidad Federal de Minas Gerais, Av. Antonio Carlos 6627, Belo Horizonte 31270-901, Brazil; cesbufmg@yahoo.com; 7 Departamento de Química, Universidad de La Serena, Av. Raúl Bitrán 1305, La Serena 1700000, Chile

**Keywords:** *Botrytis cinerea*, biofungicide, P1G10, encapsulation, natural fungicide, antifungal modeling

## Abstract

*Botrytis cinerea* is a major phytopathogen responsible for significant postharvest losses in plant-derived foods. The increasing resistance to synthetic fungicides has driven the search for sustainable alternatives, including enzyme-based biofungicides. In this study, the proteolytic fraction P1G10 from *Vasconcellea pubescens* latex was encapsulated in an alginate–chitosan (ALG-CS) matrix to improve its stability and antifungal performance. The encapsulated formulation (ALG-CS-P1G10) retained ~95% enzymatic activity after 8 h under stress conditions (37 °C, 1350 lux), compared with 67% for the free enzyme. In vitro assays demonstrated a dose-dependent inhibition of *B. cinerea* growth, with an IC_50_ value of ~11 mg/mL determined using a logistic model. At this concentration, the formulation reduced fungal adhesion by more than 80% and increased sensitivity to cell wall-disrupting agents (Congo Red and Calcofluor White), pointing to alterations in cell wall integrity. Importantly, the encapsulated system provided a more stable and sustained antifungal effect, consistent with a controlled-release mechanism. These results demonstrate that coupling enzyme stabilization with controlled release can improve the functional performance of protease-based antifungal systems, offering a promising strategy for the development of biofungicides in postharvest applications.

## 1. Introduction

*Botrytis cinerea* (teleomorph: *Botryotinia fuckeliana*) is a necrotrophic phytopathogenic fungus responsible for gray mold disease. It employs diverse infection strategies and virulence factors, including the secretion of lytic enzymes and phytotoxic metabolites, enabling infection of more than 200 plant and vegetable species. The fungus persists in the environment as conidia or mycelium and can withstand adverse conditions through sclerotia formation [1]. Moreover, more than 35 species belonging to the Botrytis genus have been identified to date, highlighting the genetic diversity and adaptability of this fungal group [2].

Infections caused by *B. cinerea* generate substantial economic losses during both preharvest and postharvest stages [3]. To control this pathogen, the agro-food industry has relied on physical, chemical, and alternative approaches. Physical methods, mainly applied during postharvest, include UV radiation and modified-atmosphere technologies. Chemical control remains the most widely used strategy and involves fungicides from diverse chemical groups, including triazoles, pyrimidines, carbamates, phosphates, phenylpyrroles, and carboxamides [4]. However, the use of chemical pesticides generates harmful effects on health, as they are toxic and are associated with endocrine, immunological, neurological [5], gastrointestinal and carcinogenic effects [6]. Moreover, the repeated use of chemical fungicides has accelerated the development of resistance in *B. cinerea* due to its genetic plasticity, rendering some compounds ineffective [1]. As an alternative, biofungicides, microorganisms or naturally derived compounds with antifungal properties have gained interest [7]. Examples include antagonistic microorganisms such as *Pseudomonas syringae*, *Bacillus subtilis*, *Bacillus amyloliquefaciens*, and *Trichoderma harzianum* [1,3]. Additionally, many natural compounds, such as saponins and flavonoids [8], alkaloids [9] and essential oils from clove (*Syzygium aromaticum*), mustard (*Brassica nigra*) [10], oregano, lavender, and rosemary, have demonstrated inhibitory effects against *B. cinerea* [11]. Plant latex is another promising natural antimicrobial source. Latex, produced by lactiferous cells in more than 20,000 plant species [12], is rich in metabolites, proteins, and enzymes involved in defense against herbivores, parasites, viruses, and fungi [13]. Its enzymatic components include alkaloids, terpenoids, phenolic compounds, oxidases, chitinases, and proteases [12]. Proteases are abundant in latex from plants such as papaya (papain) and fig (ficin), as well as in fruits including pineapple (bromelain) and kiwi (actinidin) [14]. Several cysteine proteases have shown antifungal or antiparasitic activity [15,16] and are attractive eco-friendly alternatives due to their biodegradability and low environmental impact. For example, bromelain at 0.30 μM/L inhibited *Fusarium verticillioides* growth to less than 20% [17].

Within this group, the proteolytic fraction P1G10 from *Vasconcellea pubescens* latex is composed of 14 isoenzymes, 12 of which exhibit amidase activity [18]. Free P1G10 inhibits *B. cinerea* by disrupting plasma membrane integrity, impairing conidial germination, and reducing fungal adhesion [19]. However, like other cysteine proteases, its enzymatic activity declines rapidly once extracted from its natural matrix: amidase activity decreases to 11% after 24 h at 37 °C and approaches zero after 48 h [20]. This instability limits its potential as a practical antifungal agent. The immobilization or encapsulation of bioactive substances is an option to stabilize the activity of bioactive species to carry out their application and have a practical use [21,22].

Encapsulation offers a promising strategy for stabilizing fragile bioactive compounds. Immobilizing proteases within alginate–chitosan (ALG-CS) matrices through polyelectrolyte complexation can enhance stability, preserve enzymatic activity, and allow controlled release under specific temperature and pH conditions [23,24,25]. Although this approach has enabled the development of ALG-CS-P1G10 as a biofungicide candidate, its antifungal effects on *B. cinerea* have not yet been fully characterized. In particular, a comprehensive evaluation integrating enzyme stabilization, controlled-release behavior, and quantitative antifungal assessment using kinetic modeling remains lacking.

Therefore, the objective of this study was to evaluate, under in vitro conditions, the antifungal potential of the ALG-CS-P1G10 formulation against *B. cinerea*, combining stability analysis, release behavior, and dose–response modeling to determine IC_50_ values and better understand its functional performance as an enzyme-based biofungicide.

## 2. Materials and Methods

### 2.1. Purification of P1G10 Proteolytic Fraction

All chemicals used in this work were obtained from Merck KGaA and Sigma-Aldrich (St. Louis, MO, USA). The P1G10 purification was done according to [18]. Briefly, 15.0 g of lyophilized papaya latex from *Vasconcellea pubescens* was mixed with 50 mL of the extraction buffer (0.010 M EDTA, 0.020 M Cysteine-HCl, 0.0020 M DTT and 0.60 M sodium acetate pH 5.1). The mixture was incubated with stirring for 30 min at room temperature and centrifuged for 10 min at 7000× *g*. The supernatant was loaded onto a chromatographic column with Sephadex G-10. The filtration of P1G10 was performed according to the methodology described by [19] and the protein composition and stability of the fraction were established by protein electrophoresis and enzyme activity dosage, as described by [26].

### 2.2. Stabilization of the Biofungicide P1G10 in ALG-CS Capsules

The immobilization of the fraction P1G10 in alginate and chitosan was done according to [26]. Alginate (ALG) and chitosan (CS) were mixed at a 1:1 mass ratio with 13.2 mg of P1G10 (52% relative to each polymer). The mixture was stirred at room temperature for 10 min, followed by the addition of 10 mL of ALG solution (2.5 mg/mL) at 5.0 mL/min under continuous stirring and further mixed for 10 min. The suspension was centrifuged (8000× *g*, 30 min, 14 °C), and a 5 mL aliquot of the supernatant was collected. The pellet was washed with 0.1 M acetate buffer (pH 5.1) and freeze-dried.

To evaluate the stability of the biofungicide ALG-CS-P1G10, the methodology described by [27] was followed with some modifications. For this purpose, 55–65 mg of the biofungicide ALG-CS-P1G10 were weighed in 10 mL glass vials and mixed with 5 mL of 0.1 M phosphate buffer at pH 6.8 containing 0.30 M NaCl, aiming to achieve approximately 70% protein release within 8 h [26]. Subsequently, the mixture of the biocatalyst ALG-CS-P1G10 with the saline buffer was incubated under gentle magnetic stirring at 37 °C and 1350 Lux to simulate operational conditions (environmental conditions). Samples of 1 mL of the mixture were taken at times 0, 1, 2, 4 and 8 h and then centrifuged at 2800× *g* reserving 900 μL of the supernatant from each sample. In parallel, a solution of 1 mg/mL concentration (corresponding to its IC_50_ value according to previous studies) of P1G10 was prepared by weighing 20 mg of the protein and mixing it with 20 mL of phosphate buffer. Finally, protein determination was carried out using the Lowry method and enzymatic activity determination using BOC-Ala-ONp as a substrate according to [28]. To express the enzymatic activity at each sampling time, the following expression was used:

(1) $${AE}_{t}=\frac{{AE}_{ti}}{{AE}_{t0}}\times \;100\%\;\;$$

AE_t_ represents the percentage of enzymatic activity (1), AE_ti_ is the enzymatic activity in a specific time, and AE_t0_ is the initial enzymatic activity.

### 2.3. Application of Biofungicide ALG-CS-P1G10 on Botrytis cinerea

To establish the effect that the ALG-CS-P1G10 complex produces on the *B. cinerea* strain B05.10, application conditions have been established. The strain B05.10 was grown at 22 °C and pH 5.1 in minimal medium (KH_2_PO_4_ 1.0 g/L, K_2_HPO_4_ 0.502 g/L, MgSO_4_ 7H_2_O 0.50 g/L, KCL 0.50 g/L, FeSO_4_ 7H_2_O 0.005 g/L, C_4_H_12_N_2_O_6_ 0.4 g/L, pectin 1 g/L, agar-agar 15 g/L). Furthermore, to facilitate the release of the P1G10 fraction from the encapsulation, sodium chloride was added to the minimal medium, ensuring a concentration of 0.30 M. According to previous studies, this NaCl concentration produces its release. In the same context, to evaluate whether the NaCl added to the medium itself produces some effect on the growth of the B05.10 strain, it was grown in minimal medium, in the presence and absence of 0.30 M NaCl. Then, 10 μL of spore suspension was inoculated with a concentration of 2.5 × 10^5^ in the center of 90 mm diameter Petri dish to each condition. The plates were incubated at 22 °C for 6 days, recording the growth diameter daily.

### 2.4. Effect of the Biofungicide ALG-CS-P1G10 on the Growth of Botrytis cinerea

To ensure that the observed antifungal effects were specifically associated with the active formulation, preliminary control assays were conducted using the ALG-CS matrix without P1G10. These experiments confirmed that the empty ALG-CS complex did not significantly affect the growth of *B. cinerea* under the tested conditions. Therefore, the growth inhibition observed in this study is attributed to the released P1G10 fraction. The concentration of the biofungicide ALG-CS-P1G10 that inhibits the growth of *B. cinerea* by 50% (IC_50_) was determined using the method of [29] with modifications. The amounts of biofungicide 2, 4, 6, 8, 10, and 12 mg/mL, respectively, were weighed into sterilized vials and mixed with minimal medium at 50 °C before depositing the medium on the plate. The temperature of 50 °C was selected as the lowest condition that allows adequate mixing of the active components with the culture medium prior to solidification, while preserving the proteolytic activity of P1G10. This selection is supported by previous reports indicating that P1G10 remains stable at moderately elevated temperatures during short incubation periods [19]. Subsequently, on the solidified culture medium, 10 μL of spore suspension (2.5 × 10^5^ spores/mL) was inoculated in the center of a Petri dish with a diameter of 90 mm. The plates were incubated at 22 °C for 6 days, recording the growth diameter daily. Fitting the *B. cinerea* colony diameter data as a function of time for different concentrations of the ALG-CS-P1G10 biocatalyst was performed using a simple two-parameter exponential growth model [29,30] according to the following expression:

(2) $$f\left(x\right)=ae^{bx}$$

In the model, f(x) represents the growth diameter as a function of time (x), a corresponds to the initial growth and b is the specific growth rate.

### 2.5. Application of Mathematical Models for Estimating IC_50_

To establish the IC_50_ value, four mathematical models were evaluated, Weibull (Equation (3)), Gompertz (Equation (4)), Logistic (Equation (5)), and Exponential (Equation (6)) [31,32,33,34], running non-linear regressions that allowed for determining the parameters of said models (a, b and c) to calculate the IC_50_ value according to each model:

(3) $$f\left(x\right)=a(1-e^{{-bx}^{c}})$$

(4) $$f\left(x\right)=ae^{{-ce}^{-bx}}$$

(5) $$f\left(x\right)=a{\left(1+{ce}^{-bx}\right)}^{-1}$$

(6) $$f\left(x\right)=c+ae^{bx}$$

To select the model that best represented the experimental data of the colony diameter of *B. cinerea* as a function of the concentrations of the biofungicide ALG-CS-P1G10, the coefficient of determination R^2^ (Equation (6)), the sum of squared errors SSE, was determined. (Equation (7)) and the root mean square error RMSE (Equation (8)) were determined according to the following equations [35]:

(7) $$R^{2}=\frac{\sum ({\hat{y}}_{i}-\bar{y}{)}^{2}}{\sum (y_{i}-\bar{y}{)}^{2}}$$

(8) $$SSE=\sum_{i=1}^{n}(y_{i}-{\hat{y}}_{i}{)}^{2}$$

(9) $$RMSE=\sqrt{\frac{\sum (y_{i}-{\hat{y}}_{i}{)}^{2}}{n-p}}$$

${\hat{y}}_{i}$ corresponds to the observed value, $\bar{y}$ is the average of the observed values of the dependent variable, $y_{i}$ corresponds to the predicted value, n is the total number of observations and p is the number of model parameters.

### 2.6. Adhesion Capacity of Botrytis cinerea

To determine the effect of the biofungicide ALG-CS-P1G10 on the adhesion capacity of the B05.10 strain, an assay based on [19] was performed with modifications. A spore suspension (2.5 × 10^5^ spores/mL) was incubated in minimal medium at 22 °C for 72 h in a 96-well plate under five treatments: I (Control): B05.10 + NaCl 0.30 M, II: B05.10 + P1G10 1.0 mg/mL, III: B05.10 + P1G10 1.0 mg/mL + NaCl 0.3 M, IV: B05.10 + ALG-CS-P1G10 11 mg/mL + NaCl 0.30 M and V: B05.10 + ALG-CS 3.3 mg/mL + NaCl 0.30 M. Subsequently, the residual and non-germinated spores were removed, and each well was washed three times with 200 μL of distilled water. Crystal violet (0.1% *v*/*v*, 100 µL) was added, incubated for 5 min, and then rinsed to eliminate excess dye. The absorbance was determined at 595 nm in a multiplate reader (Victor X3, Perkin Elmer 2030 workstation, New Life Scientific, Inc, Cridersville, OH, USA). The OD values are proportional to the amount of biofilm formed under the mentioned conditions.

### 2.7. Sensitivity to Cell Wall-Disturbing Agents (Congo Red and Calcofluor White)

To determine the sensitivity of *B. cinerea* to cell wall-disturbing agents, the methodology described by [36] was used. Briefly, a preparation of minimal medium was mixed with Congo red (CR, 500 μg/mL) and Calcofluor white (CFW, 500 μg/mL), respectively. Subsequently, on the solidified culture medium, 10 μL of spore suspension (2.5 × 10^5^ spores/mL) of different treatments 0 (*B. cinerea* without NaCl), I, II, III, IV and V described previously was inoculated at the center of each plate. The plates were incubated at 22 °C for 9 days, and the growth diameter of *B. cinerea* colonies was measured daily.

### 2.8. Membrane Integrity

The membrane integrity of *B. cinerea* was assessed using the modified approach of [37]. A conidia suspension (5 mL) with a concentration of 2.5 × 10^5^ was prepared in 2% m/V malt extract medium and incubated in a shaker at 22 °C and 150 rpm for 4 h under different treatment I (Control): B05.10 + NaCl 0.30 M, II: B05.10 + P1G10 1.0 mg/mL, III: B05.10 + P1G10 1.0 mg/mL + NaCl 0.3 M, IV: B05.10 + ALG-CS-P1G10 11 mg/mL + NaCl 0.30 M and V: B05.10 + ALG-CS 3.3 mg/mL + NaCl 0.30 M. All preparations were incubated for 4 h before exposure to the spores, ensuring adequate protein release under the designated salinity conditions. After incubation, the treatment-spores suspension was filtered with a 40 μm Falcon cell filter to retain the encapsulation and recover mainly spores. The spores were then pelleted by centrifugation at 7000× *g* and washed twice with 50 mM phosphate buffer (pH 7.0). Then, they were concentrated at 990 μL and stained with 10 μL of a propidium iodide solution with a concentration of 1.0 mg/mL, at 30 °C for 5 min. Finally, the spores were washed twice with 900 μL phosphate buffer and recovered by centrifugation at 16,000× *g* for 10 min, leaving a 100 μL concentrated spore suspension. The samples were observed with a light microscope equipped with an epifluorescence system (Eclipse E-200, Nikon Instruments-Japan, Tokyo, Japan).

### 2.9. Statistical Analysis

All the experiments were carried out in triplicate, two-way analysis of variance (ANOVA) (Statgraphics Plus 5.1 software, Statistical Graphics Corp., Herndon, VA, USA) was used to demonstrate significant differences among samples. Significance testing was performed using Fisher’s least significant difference (LSD), and differences were accepted at 95% significance. The Multiple Range Test (MRT) was used to assess the presence of homogeneous groups within each of the parameters analyzed.

## 3. Results

As previously reported by [26], the encapsulation process for the ALG-CS-P1G10 system was optimized, achieving an encapsulation efficiency of approximately 74% at a polymer mass ratio of 1.0. The physicochemical characterization of the resulting encapsulated complex was also established in that study, providing a validated basis for the formulation used in the present work.

### 3.1. Stability of ALG-CS-P1G10 Under Temperature and Light

**Figure 1:**
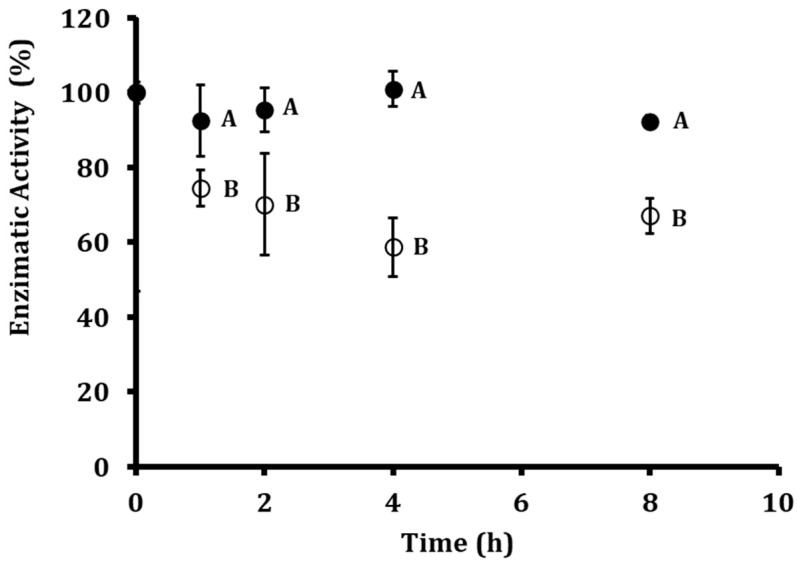
Evaluation of the stability of the biofungicide ALG-CS-P1G10 and free P1G10. Stability of the ALG-CS-P1G10 biofungicide (●) and free P1G10 protein (○) under operating conditions of light (1350 lux) and temperature (37 °C). Values are expressed as mean ± standard deviation. Different letters indicate significant differences (*p* < 0.05) according to the LSD test.

To assess the stability of the encapsulated and free proteolytic fractions, both formulations were incubated at 37 °C and 1350 lux for 8 h. This time frame was selected based on previous reports indicating that the antifungal activity of P1G10 against *Botrytis cinerea* occurs during the early stages of fungal development, making it a biologically relevant window to evaluate enzyme performance under stress conditions [19]. As shown in Figure 1, the enzymatic activity of free P1G10 declined rapidly, decreasing to 70% after 2 h, 58% after 4 h, and reaching 67% residual activity at 8 h. In contrast, ALG-CS-P1G10 maintained markedly higher stability, retaining 95% activity at 2 h, 100% at 4 h, and stabilizing at 92% after 8 h. The slight increase in enzymatic activity observed at 4 h can be attributed to the dynamic interplay between enzyme release and inactivation processes. As P1G10 is progressively released from the ALG-CS matrix into the surrounding medium, newly available active enzyme contributes to the measured activity, partially compensating for the fraction undergoing inactivation over time. This transient balance results in a temporary increase in detectable activity. In general, these results indicate that encapsulation not only protects P1G10 from rapid inactivation but also enables a sustained release profile that preserves enzymatic function under stress conditions, whereas the free form is considerably more susceptible to deactivation.

**Figure 2:**
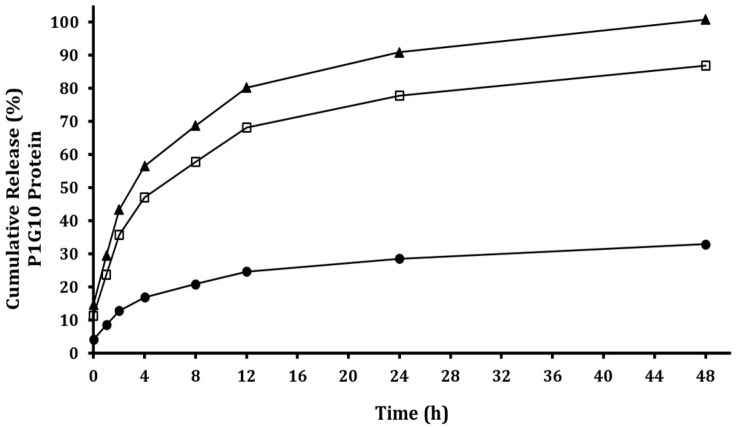
P1G10 protein release kinetics from ALG-CS-P1G10 biofungicide as a function of sodium chloride concentration. Cumulative release of P1G10 protein with NaCl concentrations: (●), 0.10 M; (◻), 0.30 M; (▲), 0.60 M. Release kinetics experiments were performed in triplicate.

In addition, the release kinetics of P1G10 from the ALG-CS matrix as a function of ionic strength are presented in Figure 2. Increasing NaCl concentration significantly enhanced protein release, reaching approximately 70% release at 0.30 M within 8 h. This behavior supports that P1G10 release is governed by electrostatic interactions within the alginate–chitosan network, where increased ionic strength weakens polymer–protein interactions, promoting enzyme diffusion into the surrounding medium. Although NaCl is known to adversely affect plant growth and metabolism, it was employed here as a proof of concept to demonstrate that ionic strength is the key factor modulating the release mechanism of the proteolytic fraction [26]. For future applications, the replacement of NaCl with more agriculturally compatible compounds should be further investigated.

### 3.2. Mathematical Models Fit and Dose–Response of ALG-CS-P1G10 Against Botrytis cinerea

**Figure 3:**
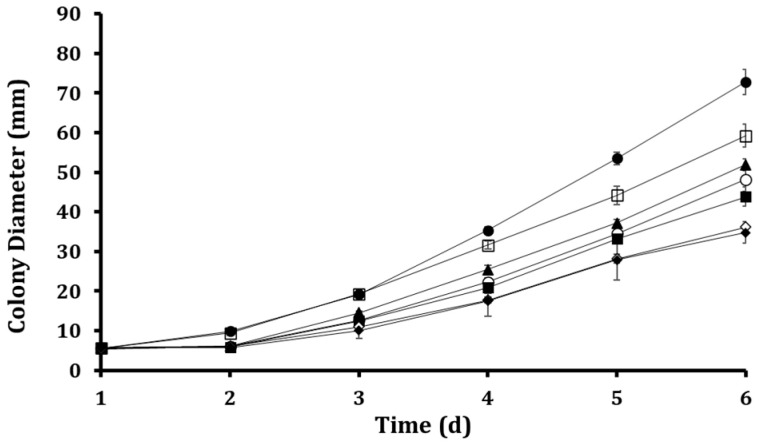
Effect of ALG-CS-P1G10 biofungicide concentration on the *Botrytis cinerea* growth in agar media. Control 0 mg/mL (●); 2 mg/mL (⧠); 4 mg/mL (▲); 6 mg/mL (○); 8 mg/mL (◼); 10 mg/mL (◇) and 12 mg/mL (◆).

The inhibitory effect of ALG-CS-P1G10 on *B. cinerea* was evaluated at concentrations ranging from 2 to 12 mg/mL on minimal media (Figure 3). The empty ALG-CS matrix did not show a significant effect on fungal growth in preliminary assays, confirming that the observed inhibition is attributable to the bioactive P1G10 fraction. As shown in Figure 3, fungal growth progressively increased over time for all conditions; however, a clear concentration-dependent inhibitory effect was observed. Higher concentrations of ALG-CS-P1G10 led to a more pronounced reduction in colony diameter, particularly from day 4 onwards, where differences between treatments became more evident. At the highest concentrations (10–12 mg/mL), fungal growth was substantially delayed compared to the control, indicating a strong antifungal effect. In contrast, lower concentrations (2–4 mg/mL) showed only moderate inhibition, with growth trends closer to the untreated control. Overall, these results demonstrate that the antifungal activity of the encapsulated system is both time- and dose-dependent, highlighting the effectiveness of P1G10 when delivered within the ALG-CS matrix.

**Figure 4:**
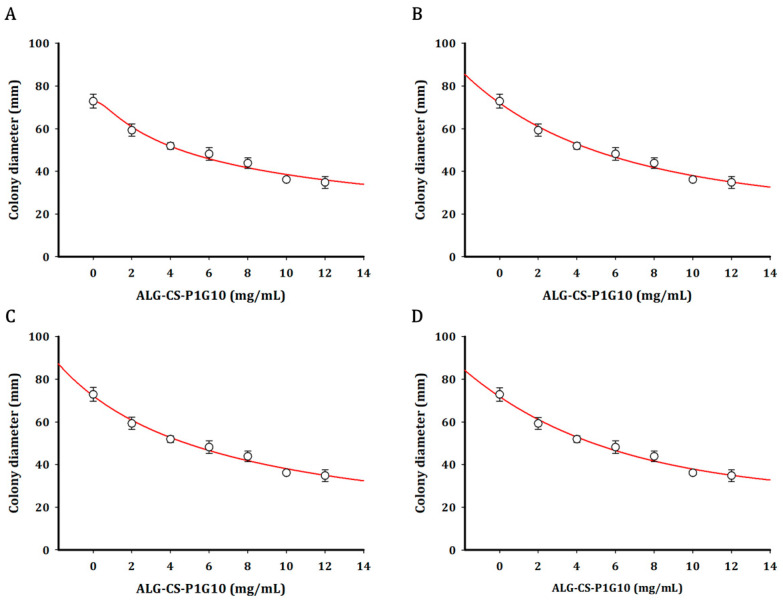
Fitting *Botrytis cinerea* growth data as a function of different concentrations of the ALG-CS-P1G10 complex using non-linear regressions for the determination of IC_50_: Weibull (**A**), Gompertz (**B**), Logistic (**C**) and Exponential (**D**).

Growth curves were fitted using Weibull, Gompertz, Logistic, and Exponential models (Figure 4). The kinetic parameters derived from exponential model fitting (Table 1) revealed a concentration-dependent decline in both the initial diameter (parameter a) and the specific growth rate (parameter b). While the control and 2 mg/mL treatments exhibited the highest initial diameters (5.665 and 5.905 mm, respectively), increasing biofungicide concentrations progressively reduced this parameter, reaching 3.581 mm at 12 mg/mL. A similar trend was observed for the specific growth rate, indicating that higher concentrations not only delay colony establishment but also slow subsequent radial expansion. Notably, the reduction in b was more pronounced at higher concentrations (10–12 mg/mL), suggesting that the biofungicide exerts a stronger effect on the dynamic phase of fungal growth rather than solely on initial development. This behavior is consistent with a sustained inhibitory effect, likely associated with the gradual release of the active proteolytic fraction from the ALG-CS matrix. All models adequately described the experimental data (R^2^ > 0.97), with relatively low SSE and RMSE values (Table 2), confirming the robustness of the fitting approach. Among them, the logistic model provided the best overall fit (R^2^ = 0.9873; SSE = 13.60; RMSE = 1.844), and was therefore selected for IC_50_ estimation. The calculated IC_50_ value (~11 mg/mL) is in good agreement with the dose-dependent inhibition observed in Figure 3, further supporting the effectiveness of the encapsulated P1G10 system.

### 3.3. Antifungal Effects of P1G10 and ALG-CS-P1G10 Treatments on Adhesion Capacity of B. cinerea

**Figure 5:**
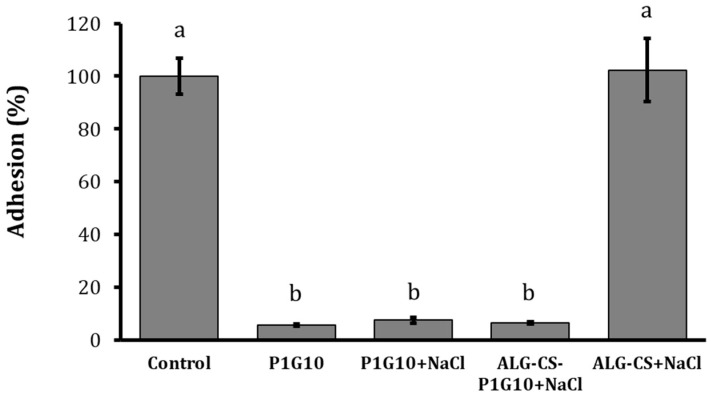
Evaluation of adhesion capacity. Effect of treatments on the adhesion capacity of *Botrytis cinerea*. I (Control): B05.10 + NaCl 0.30 M, II: B05.10 + P1G10 1.0 mg/mL, III: B05.10 + P1G10 1.0 mg/mL + NaCl 0.3 M, IV: B05.10 + ALG-CS-P1G10 11 mg/mL + NaCl 0.30 M and V: B05.10 + ALG-CS 3.3 mg/mL + NaCl 0.30 M. The values of the adhesion capacity are expressed as mean ± standard deviation. Different letters indicate significant differences (*p* < 0.05) according to the LSD test.

The biofilm formation and capacity adhesion are considered an important virulence attribute of pathogenic fungi [36]. The effects of P1G10 and P1G10 + NaCl treatments on adhesion capacity of *B. cinerea* were shown in Figure 5. The adhesion capacity of *B. cinerea* mycelia growth was evaluated in polystyrene microtiter plates after 72 h of incubation at 22 ± 1 °C. Mycelia were stained with crystal violet and subjected to a detachment force to assess the impact of P1G10 and P1G10 + NaCl treatments on the adhesion of *B. cinerea* to the polystyrene surface. The results reveal a significant reduction in the adhesion capacity of *B. cinerea* with P1G10 (94.4%) compared to the control and the complex without the ALG-CS, a similar trend was observed when *B. cinerea* was incubated with P1G10 + NaCl or ALG-CS-P1G10 biofungicide with inhibition of the 92.5% and 93.4 respectively. These findings indicate that the proteolytic activity of P1G10—whether free or released from the encapsulated matrix—disrupts *B. cinerea* attachment to abiotic surfaces.

### 3.4. P1G10 and ALG-CS-P1G10 Treatments Damage Cell Wall and Membrane Integrity in B. cinerea

**Figure 6:**
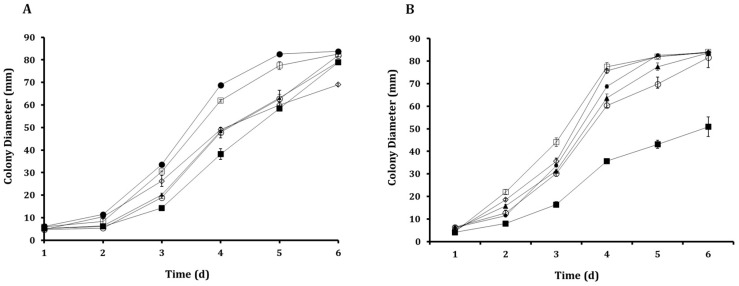
Effects of ALG-CS-P1G10 on sensitivity of *B. cinerea* grown on agar medium supplemented with Congo Red (CR) (**A**) and Calcofluor White (CFW) (**B**). Symbols represent the following treatments: B05.10 control (●), B05.10 + NaCl 0.30 M (⧠), B05.10 + P1G10 1.0 mg/mL (▲), B05.10 + P1G10 1.0 mg/mL + NaCl 0.3 M (○), B05.10 + ALG-CS-P1G10 11 mg/mL + NaCl 0.30 M (◼) and B05.10 + ALG-CS 3.3 mg/mL + NaCl 0.30 M (◇). Values are presented as mean ± standard deviation.

The results on growth of *B. cinerea* in a minimal medium prepared with Congo Red under the presence of P1G10 and P1G10 + NaCl treatments were shown in Figure 6A. On day 3, the control (*B. cinerea* without NaCl) exhibited the largest growth diameter (33 mm), compared with *B. cinerea* grown in the presence of NaCl (30 mm). This trend remained evident on days 4 and 5, whereas no significant differences were detected on day 6. Likewise, when *B. cinerea* was exposed to the ALG-CS-NaCl complex, radial growth decreased further to 26 mm, indicating increased sensitivity to Congo Red. Furthermore, P1G10, applied both with and without NaCl, reduced the radial growth of *B. cinerea* relative to the control, with each treatment yielding approximately 18 mm of growth and thus indicating a comparable increase in sensitivity under both conditions. Consistently, on days 4 and 5, reduced radial growth relative to the control was observed in cultures treated with either the ALG-CS complex or P1G10 (with or without NaCl). During this period, the presence of NaCl did not significantly modify the antifungal effects exerted by either treatment on *B. cinerea*. ALG-CS-P1G10 produced the strongest inhibition, particularly during days 3 to 5, suggesting increased CR sensitivity resulting from damage to cell-wall integrity. Figure 6B illustrates radial growth in minimal medium supplemented with Calcofluor White after P1G10 and P1G10 + NaCl treatments. In the presence of Calcofluor White (CFW), NaCl + ALG-CS-P1G10 again exhibited the highest inhibitory effect, restricting radial growth to 35 mm on day 4 (Figure 6B). The ALG-CS complex + NaCl allowed growth up to 75 mm, whereas P1G10 (with or without NaCl) reduced growth to 60–63 mm. These results show that the encapsulated formulation increases the susceptibility of *B. cinerea* to CFW more strongly than free P1G10.

**Figure 7:**
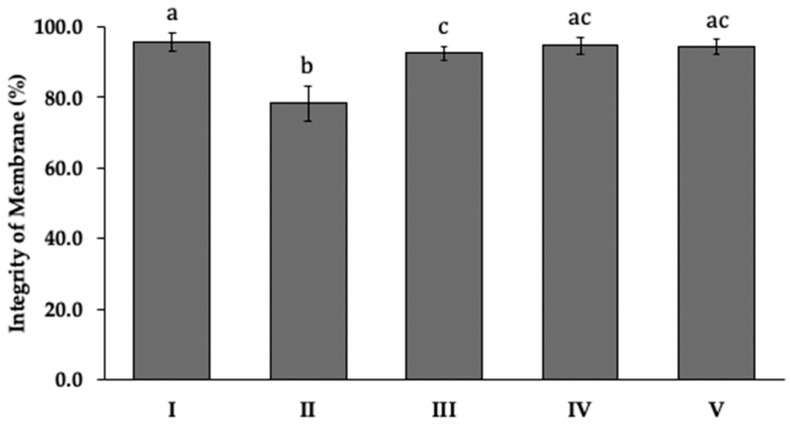
Evaluation of membrane integrity. Effect of treatments on the integrity of membrane of *B. cinerea*. I (Control): B05.10 + NaCl 0.30 M, II: B05.10 + P1G10 1.0 mg/mL, III: B05.10 + P1G10 1.0 mg/mL + NaCl 0.3 M, IV: B05.10 + ALG-CS-P1G10 11 mg/mL + NaCl 0.30 M and V: B05.10 + ALG-CS 3.3 mg/mL + NaCl 0.30 M. The values of the membrane integrity are expressed as mean ± standard deviation. Different letters indicate significant differences (*p* < 0.05) according to the LSD test.

Propidium iodide (PI) was used to assess the occurrence of loss of membrane integrity in *B. cinerea* with each encapsulation treatment (Figure 7). Significant loss of membrane integrity occurred only in the presence of free P1G10, decreasing to 78% without NaCl and 92% with NaCl. In contrast, neither ALG-CS-P1G10 nor the ALG-CS complex alone produced significant membrane damage, both maintaining integrity around 94%. These results suggest that NaCl may partially mitigate membrane disruption, and that the encapsulated form exerts a milder direct effect on membranes compared with the free enzyme.

## 4. Discussion

Enzyme fractions such as P1G10 are intrinsically unstable and rapidly lose activity under physiological or environmental stress. Improving their stability is therefore essential for both pharmacological and agro-biotechnological applications, where sustained enzymatic function directly determines efficacy. Previous work demonstrated that complexing P1G10 with β-cyclodextrin preserves enzymatic activity over extended periods, maintaining 23% activity from day 2 to day 16, compared with a dramatic loss of activity in its free form after 48 h at 37 °C [20]. Although the time frame evaluated in the present study was shorter, this is consistent with the biological window during which *B. cinerea* is most susceptible—early germination. Under our release conditions, approximately 70% of P1G10 is released between 4 and 8 h, coinciding with the period in which the pathogen initiates infection. This behavior indicates that the release mechanism is primarily governed by electrostatic interactions. The protein is entrapped within a polyelectrolyte network formed by chitosan (positively charged amino groups) and alginate (negatively charged carboxyl groups). Upon increasing ionic strength, ions from NaCl screen these electrostatic interactions, weakening both polymer–polymer and polymer–protein associations. This shielding effect reduces the cohesion of the network and facilitates the diffusion of the protein into the surrounding medium [26].

Comparison of our findings with previous studies indicates that the biofungicide ALG-CS-P1G10 maintains higher enzymatic activity than the free form of P1G10. Similar improvements in enzyme stability have been reported for encapsulated systems. For example, L-Asparaginase II encapsulated in chitosan (CS)–tripolyphosphate (TPP) nanoparticles retained 64% enzymatic activity after 48 h, whereas the free enzyme retained only 11% activity, demonstrating that encapsulation enhances enzyme stability [37]. Likewise, papain immobilized in Fe_3_O_4_/SF magnetic nanoparticles maintained ~85% residual activity after 28 days, compared with 55% for the free enzyme [38]. These findings support the immobilization of the proteolytic fraction P1G10 as a strategy to enhance its stability and antifungal functionality. P1G10 has previously shown strong antifungal activity against *B. cinerea*, inhibiting mycelial growth and adhesion while disrupting the plasma membrane and cell wall [19].

Importantly, control experiments with the ALG-CS matrix alone did not show significant antifungal activity, indicating that the observed effects are primarily associated with the proteolytic activity of P1G10 rather than the carrier system. This supports the role of the alginate–chitosan matrix as a stabilizing and controlled-release platform rather than an active antifungal agent. In the present study, fungal growth-diameter data obtained at different concentrations of ALG-CS-P1G10 were successfully fitted to four mathematical models with acceptable statistical performance. The predicted IC_50_ values were similar for the Gompertz, Logistic, and Exponential models (≈11 mg/mL), whereas the Weibull model predicted a slightly higher value (11.59 mg/mL). Previous studies have shown that IC_50_ estimates may vary depending on the mathematical model used [39]. In agronomic studies evaluating fungicides such as epoxiconazole against *Sclerotinia sclerotiorum*, the model and software used to fit dose–response curves significantly influenced IC_50_ values [40]. Accordingly, transparent criteria for model selection are essential. In our case, model selection was supported by R^2^, SSE, and RMSE values reported in Section 3.2 (Figure 3).

The IC_50_ value obtained for ALG-CS-P1G10 (~11 mg/mL) is higher than that reported for the free P1G10 fraction (1 mg/mL against *B. cinerea* [19]). This difference likely reflects the controlled release of P1G10 from the alginate–chitosan matrix, which results in a gradual antifungal effect as the enzyme becomes available. P1G10 from the alginate-chitosan matrix, which results in a gradual antifungal effect as the enzyme becomes available. It is important to note that this comparison is based on equivalent inhibitory responses (IC_50_ values) rather than nominal concentrations, as the ALG-CS-P1G10 system contains approximately 74% P1G10 and releases the enzyme progressively depending on the ionic strength of the medium. Therefore, the observed antifungal activity is governed by the fraction of bioavailable enzyme over time, rather than the total amount initially encapsulated. Comparable IC_50_ values have been reported for natural antifungal compounds such as eugenol derivatives [41] and polygodial [42], which show IC_50_ values near 0.5 mg/mL when applied in free form. Other biological systems show considerably higher IC_50_ values; for example, the biofungicide *Penicillium chrysogenum* VKM F-4876D exhibits an IC_50_ of approximately 2500 mg/mL against *B. cinerea* [43]. In contrast, chemical fungicides such as difenoconazole and tebuconazole inhibit fungal growth at concentrations close to 1 mg/mL. Notably, combining the biofungicide with these fungicides (1–0.5 mg/mL) resulted in 85% inhibition of fungal growth, suggesting that such combinations could reduce chemical fungicide usage. Integrating biofungicides with conventional fungicides may therefore reduce chemical load and associated health risks while maintaining antifungal efficacy [5].

Mathematical modeling of microbial growth is essential for predicting kinetic behavior, particularly in applications related to food quality and safety [30]. In this study, increasing concentrations of ALG-CS-P1G10 progressively reduced the growth of *B. cinerea*, decreasing both initial growth and, in most cases, the specific growth rate (Table 2). Similar trends have been reported for other antifungal compounds. For instance, betel leaf essential oil (*Piper betle* L.) reduced the specific growth rate of *Aspergillus flavus* and extended the lag phase [44]. Likewise, increasing concentrations of propionic acid reduced the growth rate of *B. cinerea* and prolonged the lag phase when modeled using modified Gompertz, Logistic, and Baranyi–Roberts equations [29]. The two-parameter exponential model used in this study showed strong predictive performance (R^2^ = 0.9819–0.9873; RMSE = 1.844–2.205), comparable to the more complex Baranyi–Roberts model reported by [44] (R^2^ = 0.785–0.999; RMSE = 0.7–6.1) [44]. Despite its simplicity, the exponential model adequately described the experimental data, indicating that additional model complexity did not significantly improve predictive accuracy.

Adhesion of *B. cinerea* to plant surfaces is a key virulence factor and a critical step in host penetration and colonization [45]. ALG-CS-P1G10 significantly reduced fungal adhesion, consistent with previous observations obtained with the free form of P1G10 [19]. Similar reductions in adhesion have been reported following treatment with metal inhibitors such as copper and iron, which may interfere with the synthesis of glycoproteins involved in fungal virulence [35,45,46]. Proteomic studies have identified more than 200 infection-related proteins in *B. cinerea*, including cell wall-degrading enzymes, proteases, and polysaccharide-modifying proteins [47]. Mutants lacking genes associated with these proteins often show normal growth but markedly reduced virulence, supporting the importance of cell wall remodeling and adhesion in the infection process [36]. The fungal cell wall, composed mainly of mannoproteins, β-glucans, and chitin, is essential for maintaining cellular integrity and resistance to environmental stress [47,48]. Cell-wall-targeting agents such as Calcofluor White (CFW) and Congo Red (CR) disrupt cell-wall synthesis and induce morphological alterations that compromise fungal viability [49,50]. The antifungal activity of ALG-CS-P1G10 appears to be primarily associated with progressive cell wall destabilization rather than direct membrane disruption, which was less pronounced in the encapsulated system compared to free P1G10.

Our results indicate that the biofungicide ALG-CS-P1G10 induces alterations in the fungal cell wall that increase susceptibility to CFW [49,51]. Studies on *B. cinerea* mutants lacking the bcpmr1 gene, encoding a Ca^2+^/Mn^2+^-ATPase, have shown increased sensitivity to membrane-disrupting agents such as CFW, CR, and caffeine [36]. In addition, Refs. [51,52] reported that treatment with CFW and CR reduced mycelial growth and conidial production in *Sporothrix globosa*. Similarly, disruption of glycosylation enzymes such as O-mannosyltransferases (PMTs) results in reduced growth, increased sensitivity to cell-wall stressors, and decreased virulence [53].

Together, these findings suggest that the antifungal activity of ALG-CS-P1G10 results from the gradual release of P1G10, which compromises fungal cell-wall integrity. The increased sensitivity of *B. cinerea* to CFW and CR following treatment further supports the hypothesis that the proteolytic fraction released from the alginate-chitosan matrix disrupts normal fungal growth [19]. Moreover, damage to the fungal cell wall may lead to leakage of intracellular components, as previously observed when mycelia were exposed to increasing concentrations of boron [36]. The reduced membrane damage observed when P1G10 was combined with NaCl suggests that osmotic stabilizers may partially protect the fungal membrane, consistent with reports showing that salts such as KCl can partially restore growth and morphology in *B. cinerea* mutants [53]. The sustained exposure to the released enzyme may overwhelm these adaptive responses, ultimately leading to reduced fungal growth, impaired adhesion, and decreased virulence potential. The encapsulation strategy therefore not only improves enzymatic stability but also provides a controlled-release antifungal system capable of exerting prolonged inhibitory effects on *B. cinerea*.

Chitosan (CS) is a positively charged, non-toxic, biocompatible, and biodegradable polymer [23], while sodium alginate (ALG) is a negatively charged polymer with similar properties, widely used in encapsulation and controlled-release systems [24]. The plant-derived proteolytic fraction P1G10 further supports the potential of this system as an environmentally friendly alternative to synthetic fungicides. Accordingly, the ALG-CS-P1G10 complex represents a promising strategy for controlling *B. cinerea* growth. Future applications may involve environmentally compatible ions to trigger P1G10 release under field conditions; however, this approach is currently under intellectual property protection.

## 5. Conclusions

The proteolytic fraction P1G10 was successfully stabilized through encapsulation in an alginate–chitosan matrix, generating the biofungicide ALG-CS-P1G10 with enhanced enzymatic stability. The formulation retained approximately 95% of its activity after 8 h under stress conditions, significantly outperforming the free enzyme. In vitro assays demonstrated dose-dependent inhibition of *B. cinerea*, with an IC_50_ value of ~11 mg/mL, and revealed significant effects on fungal adhesion and increased sensitivity to cell wall-disturbing agents, indicating alterations in cell wall integrity. Although membrane damage was less pronounced than with free P1G10, the encapsulated system provided a more stable and sustained antifungal effect.

Overall, these findings support the potential of ALG-CS-P1G10 as a controlled-release, enzyme-based biofungicide. However, further studies under field and postharvest conditions, as well as toxicological evaluations, are required to validate its practical applicability.

## Figures and Tables

**Table 1:** Kinetic parameters of the growth of *Botrytis cinerea* under different concentrations of the biofungicide ALG-CS-P1G10. Determination of the initial growth parameter (parameter a) and specific growth rate (parameter b) according to the exponential model as a function of time for the different concentrations of biofungicide ALG-CS-P1G10. The values of the kinetic parameters (a and b) are expressed as mean ± standard deviation. Different letters indicate significant differences (*p* < 0.05) according to the LSD test.

Biofungicide Concentration	Model Coefficient		Statistical Parameters		
ALG-CS-P1G10 (mg/mL)	a	b	R^2^	SSE	RMSE
0	5.665 ^A^ ± 0.215	0.4320 ^A^ ± 0.010	0.9815	64.84	4.026
2	5.905 ^A^ ± 0.506	0.3909 ^BC^ ± 0.020	0.9790	45.78	3.383
4	4.223 ^B^ ± 0.281	0.4234 ^AB^ ± 0.014	0.9838	27.49	2.622
6	3.690 ^B^ ± 0.309	0.4331 ^A^ ± 0.012	0.9879	17.58	2.097
8	3.798 ^B^ ± 0.358	0.4145 ^ABC^ ± 0.022	0.9818	21.94	2.342
10	3.644 ^B^ ± 0.249	0.3891 ^C^ ± 0.012	0.9827	13.49	1.837
12	3.581 ^B^ ± 1.153	0.3967 ^BC^ ± 0.055	0.9746	18.82	2.169

**Table 2:** Mathematical models for IC_50_ estimation. The letters a, b and c are parameters of the Weibull, Gompertz, Logistic and Exponential mathematical models. R^2^ represents coefficient of determination, SSE is residual sum of squares and RMSE corresponds to mean square error.

Model	Equation	Parameters	IC_50_ (mg/mL)	R^2^	SSE	RMSE
Weibull	$f\left(x\right)=a(1-e^{{-bx}^{c}})$	a = 72.54 b = 2.661 c = −0.5463	11.59	0.9819	19.44	2.205
Gompertz	$f\left(x\right)=ae^{{-ce}^{-bx}}$	a = 20.40 b = 0.07070 c = −1.261	10.98	0.9862	14.79	1.923
Logistic	$f\left(x\right)=a{\left(1+{ce}^{-bx}\right)}^{-1}$	a = 7.502 b = 0.01100 c = −0.8960	10.98	0.9873	13.60	1.844
Exponential	$f\left(x\right)=c+ae^{bx}$	a = 46.50 b = −0.1306 c = 25.30	10.94	0.9851	16.01	2.001

## Data Availability

The original contributions presented in the study are included in the article; further inquiries can be directed to the corresponding authors.

## Abbreviations

The following abbreviations are used in this manuscript:

ALG	Alginate
CS	Chitosan
CR	Congo Red
CFW	Calcofluor White
CDA	Chitin deacetylases
PI	Propidium iodide
TPP	Tripolyphosphate
